# De-Implementing Opioids for Dental Extractions (DIODE): a multi-clinic, cluster-randomized trial of clinical decision support strategies in dentistry

**DOI:** 10.1186/s13012-023-01262-7

**Published:** 2023-02-10

**Authors:** Jan Gryczynski, Shannon Gwin Mitchell, Stephen E. Asche, Anjali R. Truitt, Donald C. Worley, D. Brad Rindal

**Affiliations:** 1 Friends Research Institute, Baltimore, MD USA; 2 HealthPartners, Minneapolis, MN USA

**Keywords:** Opioids, Dentistry, De-implementation, Clinical decision support

## Abstract

**Background:**

Opioid pain relievers are commonly prescribed following dental extractions, but evidence shows that non-opioid analgesics often provide adequate pain relief with fewer risks. The current study examined clinical decision support (CDS) as a tool for de-implementing opioid prescribing in dentistry.

**Methods:**

This prospective, cluster-randomized trial examined CDS for dental pain management at 22 HealthPartners Dental Group clinics in Minnesota. Dental providers (*n* = 49) were randomized to deliver care using CDS, CDS with patient education materials (CDS-E), or standard practice (SP). Randomization was stratified by provider type (dentist vs. oral surgeon) and baseline opioid prescribing volume. Patient records of dental extractions were examined for January 2019 through May 2021, representing a 12-month baseline and 15-month intervention period (*N* = 12,924). Opioid prescription at the visit (no vs. yes) was the primary outcome. Data were analyzed using generalized linear mixed models, adjusting for patient sex and age, extraction complexity, and baseline prescribing strata (volume and provider type).

**Results:**

Patients were 56.2% female, with a mean age of 46.7 (SD = 20.0) years. Providers were 8% oral surgeons, 57% female, and with a mean age of 43.7 (SD = 11.2) years. There were significant decreases in opioid prescribing during the study (*P* < 0.001), representing a continuation of pre-existing trends to reduce opioid prescribing in these dental practices. There were no significant differences in opioid prescribing between CDS and SP (OR = 1.29; 97.5% CI = 0.93, 1.79; *P* = 0.08), or CDS-E and SP arms (OR = 1.27; 97.5% CI = 0.86, 1.79; *P* = 0.18). The direction of the association favored greater reductions in opioid prescribing in the SP arm. Despite training and implementation support, utilization of the CDS was low, particularly among oral surgeons, who were significantly more likely than other dentists to prescribe opioids. Among non-oral surgeon providers with the opportunity to access it, CDS utilization was not significantly associated with opioid prescribing.

**Conclusions:**

Equipping dentists with CDS resources, whether alone or accompanied by patient education materials, did not accelerate reductions in opioid prescribing beyond those observed in standard practice. Strategies are needed to enhance CDS utilization for patient care and safety surrounding analgesia following dental extractions.

**Trial registration:**

Clinicaltrials.gov, NCT03584789.

**Supplementary Information:**

The online version contains supplementary material available at 10.1186/s13012-023-01262-7.

Contributions to the literature

- Opioids are often prescribed for pain management following dental extractions, despite evidence that non-opioid alternatives are often effective in managing post-extraction pain.
- The current study focuses on de-implementation of the common and potentially harmful practice of opioid prescribing in dentistry.
- Equipping dentists with clinical decision support resources, whether alone or paired with patient education materials, did not reduce opioid prescribing compared to standard practice.
- This study adds to the literature on de-implementation of opioid prescribing, clinical decision support strategies, and changing clinical practices in dentistry.

## Introduction

Opioid overdose deaths are a continuing public health crisis in the USA. Drug overdose fatalities exceeded 100,000 people over a 12-month period ending in April 2021, with the majority of these deaths attributable to opioids [1]. This problem has reached such extremes that it is thought to contribute significantly to declines in life expectancy at a population level [2, 3]. While overdose deaths are increasingly driven by synthetic opioids in the illicit drug supply, the origins of this crisis lay in years of overprescribing opioids for pain by healthcare providers. The past decade has seen major efforts to reduce opioid prescribing as the healthcare system comes to terms with its role in shaping the crisis [4, 5]. Nevertheless, despite declining substantially from their peak, prescription opioid analgesics continue to contribute to overdose deaths [6].

### Role of dentistry in opioid prescribing

Dentistry has played a role in the overprescribing of opioids in the USA [7–9]. Between 2011 and 2015, approximately half of opioid prescriptions written by dentists for adults exceeded recommendations for acute pain in terms of duration, while a quarter exceeded recommendations in terms of dose [7]. Opioid analgesics prescribed pursuant to dental procedures are frequently a source of first opioid exposure for adolescents and young adults [10]. Notably, opioid prescriptions among dentists in the USA are many-fold higher than among dentists in England, with one study showing that opioids accounted for 22.3% of dental prescriptions in the USA, compared 0.6% of dental prescriptions in England [11]. Research has found that opioid prescribing in dentistry is associated with serious adverse outcomes as well as persistent opioid use [12].

The reliance on opioids in dentistry rests on scant evidence. Clinical trials have found that combining non-steroidal anti-inflammatory drugs (NSAIDs) and acetaminophen provides comparable analgesia to opioids for dental extractions, without the attendant risks of misuse and adverse outcomes [13, 14]. Thus, in most clinical circumstances surrounding post-extraction pain, the NSAID-acetaminophen combination represents a safe and effective alternative to opioids. Nevertheless, although opioid prescribing in dentistry has been decreasing in recent years [8, 15] (consistent with trends in the US healthcare system as a whole), many dentists continue to prescribe opioids routinely [16].

### Clinical decision support as an opioid de-implementation tool

At a basic level, the decision of whether to prescribe an opioid to a given patient rests on the provider. Therefore, provider-level interventions are needed to change what has become an entrenched prescribing practice. One approach to changing provider behavior is the deployment of a clinical decision support (CDS) system. CDS offers real-time guidance and resources to healthcare providers to facilitate the delivery of the best evidence care for a specific patient. However, because opioid prescribing for post-extraction pain became very common, many patients may come to the dentist expecting that they will receive opioid analgesics to manage their pain [17]. Thus, it may also be important to educate patients about the risks of opioids and the effectiveness of the alternatives.

In order to align dental practices with current best evidence guidelines, it is necessary to de-implement the widespread use of opioids, while implementing the use of non-opioid approaches to managing post-extraction pain using the alternative of the NSAID-acetaminophen combination. De-implementation research shares many characteristics with traditional implementation science, but there are aspects that differ in the framing of research questions and de-implementation strategies [18]. In the current context, the goal of de-implementation is not to eliminate the use of opioids for post-extraction pain, as opioids may be appropriate in certain cases (e.g., more complex extractions, intolerability of NSAIDs). Rather, the goal is to reduce the overreliance on opioids, which are not necessary for most tooth extractions. A CDS has the potential to aid in opioid de-implementation by providing guidance for providers and patients, informing prescribing decisions and care recommendations that can be personalized to the individual’s unique medical history and risk factors while reflecting the best available evidence.

In the current study, we sought to test the effectiveness of CDS with and without patient education, relative to usual care, in de-implementing opioid prescribing for dental extractions at a large multi-clinic dental system in the Midwest.

## Methods

### Design

**Fig. 1:**
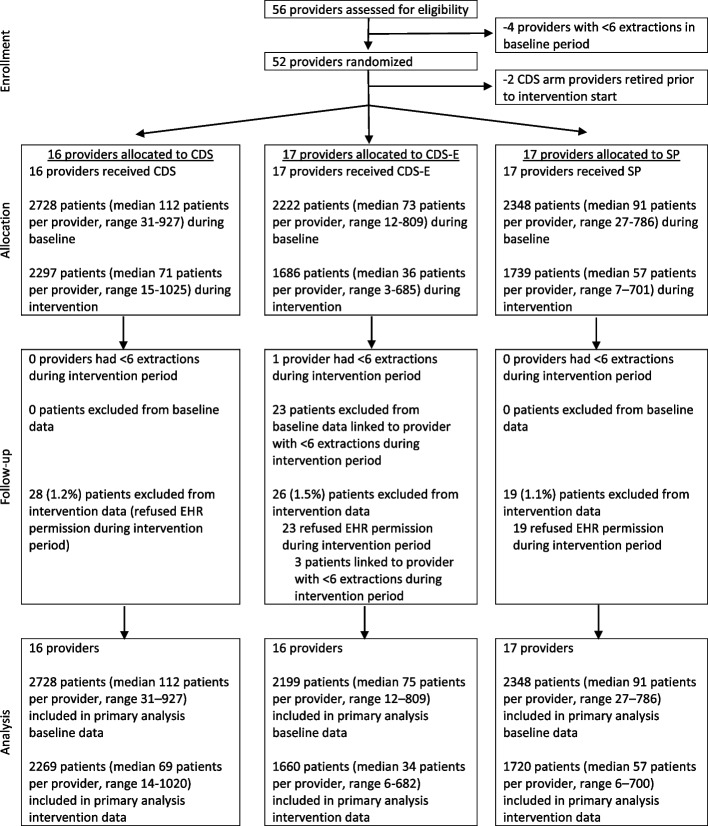
Study flow CDS, clinical decision support; CDS-E, clinical decision support with patient education; SP, standard practice

The study was a prospective, cluster-randomized trial of CDS to de-implement opioid prescribing. Due to the impracticality of randomizing at the patient level, randomization occurred at the provider level, whereby dental providers were randomly assigned on a 1:1:1 basis to one of three study arms: standard practice (SP), clinical decision support (CDS), or clinical decision support augmented with patient educational materials (CDS-E). Randomization was stratified by provider type (dentist vs. oral surgeon) and providers’ volume of opioid prescribing in the baseline period. The study statistician (SA) generated the randomization sequence. Fifty providers were randomized, with 1 subsequently excluded due to a very low volume of extractions during the intervention period. The analysis sample consisted of 49 providers with 12,924 unique patients. Our hypothesis was that the CDS and CDS-E arms would show greater reductions in opioid prescribing compared to standard practice. The outcome of whether or not an opioid was prescribed was assessed at the patient level, and patients were blind to their provider’s study arm. Figure 1 depicts the study flow. The study protocol and methodological details have been previously reported [19]. The HealthPartners Institutional Review Board approved the study.

### Setting

This study was conducted at 22 HealthPartners dental clinics in Minnesota. HealthPartners is the largest consumer-governed non-profit healthcare organization in the USA. It was an early adopter of health information technology and all dental clinics use an electronic health record (EHR) that integrates dental and medical records. Many providers practiced across multiple clinics during the study period, with only 6 of 49 providers completing all of their extraction encounters at a single clinic.

### Eligibility

The CDS was considered a quality improvement initiative of the HealthPartners Dental Group, and the project had support and approval from leadership within the organization. The local IRB considered this approval as an alternative to individual, written informed consent for dentists enrolled in the study. Thus, all eligible dentists and oral surgeons at HealthPartners were included. Dentists and oral surgeons at HealthPartners who performed at least 6 dental extractions in the year prior to launching the trial were eligible for inclusion in the study. Patients were included in the study if they were age 16 or older and had a permanent tooth extraction that was performed by an eligible provider at HealthPartners. HealthPartners routinely conducts data-driven quality improvement initiatives and research on services and patient outcomes. All patients at HealthPartners are given the opportunity to opt out of having their data used for research. Patients who had opted out of research still saw their provider as they normally would, but their data were not used in the current study.

### Study arms

Dentists and oral surgeons were given access to the CDS according to the arm to which they were randomized. All providers at HealthPartners were made aware of the project, and there was no blinding. Providers in the SP arm were not given access to the CDS module, whereas the CDS was accessible for providers in the CDS and CDS-E arms. Patients were thus exposed to the intervention to which their provider was randomized. Providers in the CDS and CDS-E arms were informed about their assignment and trained on the purpose of the CDS, what health information it incorporated, and how to access the CDS through written communication with an opportunity for clarification.

#### Standard practice (SP)

Providers in the SP arm did not have access to the CDS in the EHR. The SP arm represents the control condition in the study.

#### Clinical decision support (CDS)

Providers in the CDS arm were given access to the CDS at the point-of-care through the EHR, which was accessible to providers who were seeing patients for a dental extraction, with a highlighted link in the EHR when a patient had a treatment plan involving an extraction. The CDS content was developed by the research team and content experts to provide guidance on pain management recommendations for dental extractions. Prior to deploying the CDS, the study team conducted observations of 15 dental extractions at HealthPartners to determine provider workflow related to analgesic decision-making, including review of the EHR and discussions with the patient, in order to optimize the placement and timing of the CDS within the EHR. The CDS was personalized for the patient to display potential medication interactions with commonly recommended analgesics as identified in Micromedex [20], flagged relevant health conditions that may have a bearing on pain management decisions and risk for opioid misuse (including history of substance use disorders, history of overdose, current naloxone prescription), and provided automated access to the state’s prescription drug monitoring program (PDMP). The CDS then provided guidance regarding analgesia options to consider recommending based on the available evidence with the overarching goal of providing personalized healthcare. The CDS was designed to simplify decision-making by synthesizing this relevant information in a single interface within the EHR.

#### Clinical decision support with patient education (CDS-E)

Providers in the CDS-E arm had the same access to the CDS as providers in the CDS arm. In addition, the front desk printed an educational handout at check-in for patients with a treatment planned extraction. This handout included information comparing opioid and non-opioid options for analgesia on risks, benefits, and effectiveness. The intention of the handout was to equip patients with information about medication options and prompt a discussion with the provider about needs, goals, and preferences. Although this type of enhanced patient-provider communication may not be very common in dentistry in the USA, it was thought that it could lead to more patient-centered care and improve patients’ acceptance of their analgesia plan. Furthermore, although patient information and decision aids are sometimes used in dentistry, examination of the existing materials at HealthPartners found that they did not provide information comparing the efficacy, safety, and side effects of the various analgesics. The handout sought to normalize the experience of some limited discomfort, set expectations, and provide tips for managing post-extraction pain (e.g., ice) and guidance for when to contact providers if they needed additional help in managing their post-extraction pain.

### Study endpoint and measures

Data were derived from the EHR for each extraction encounter. The primary outcome of interest was whether an opioid was prescribed at the extraction encounter. Patient and provider characteristics were also obtained from the EHR, as was provider utilization of the CDS for each extraction encounter.

The study period spanned January 2019 through May 2021, which included a 12-month baseline (pre-intervention) period and a 15-month intervention period. The intervention period was originally planned to be 12 months, but was extended in response to the disruptions in services and delays caused by the COVID-19 pandemic. The analysis sample consisted of patients ages 16 and older seen by 49 participating providers for a dental extraction. The patient’s first extraction encounter within the study period was used as the analytical record, for a total of *N* = 12,924 patients (*N* = 7275 unique patients in the baseline period, *N* = 5649 unique patients in the intervention period).

### Statistical analysis

A priori power analyses were based on 2018 data from HealthPartners and estimated 6900 patients ages 16 and older receiving extractions in each of the baseline and intervention periods seen by 51 providers (slightly more than the final analysis sample). In 2018, 40% of tooth extraction encounters included an opioid prescription, and the provider-level intraclass correlation for opioid prescribing was estimated at 0.3. Under these assumptions about HealthPartners providers and patients, and *α* = 0.05, the study had 80% power to detect a differential change of 23% from baseline to intervention periods when comparing CDS or CDS-E to SP arms.

Data were analyzed using generalized linear mixed models for binary data (binomial family with logit link function; alternatively known as mixed effects logistic regression). The dependent variable was extraction-level opioid prescription (no vs. yes). Explanatory variables include Time (baseline vs. intervention), Arm (CDS vs. CDS-E vs. SP), and the Arm × Time interaction (representing the effect of interest). Analyses were adjusted for patient sex and age, extraction complexity (simple vs. complex), and baseline prescribing strata (dentist vs. oral surgeon; baseline opioid prescribing volume). Models included a random intercept for provider to account for the cluster design. Comparisons were made from the baseline to the intervention period within each study arm, while the primary effect of interest was examined as the differential change between CDS and CDS-E arms in comparison to the SP arm. Contrasts of interest were examined using odds ratios, model-based predicted probabilities, and their confidence intervals (95% confidence intervals for baseline vs. intervention period comparisons within study arm; 97.5% confidence intervals for comparing each of the two intervention arms against SP with respect to differential change. The 97.5% confidence interval was used for the primary endpoint in order to address multiple comparisons across three study arms). Analyses were conducted on an intent-to-treat basis. To give additional context for the findings, utilization of the CDS was examined descriptively.

In addition to the primary analysis, post hoc analyses were conducted excluding oral surgeons (due to their having more opioid prescriptions and lower utilization of the CDS). The association between providers’ CDS utilization and opioid prescribing at the extraction visit was examined among providers in the CDS and CDS-E arms (i.e., those who had the opportunity to use the CDS). These post hoc analyses were conducted using a similar analytical strategy as used for the primary analysis, except that it excluded oral surgeons due to their minimal utilization of the CDS and included an interaction term between extraction complexity and CDS utilization.

## Results

### Patient characteristics

Table 1 shows the patient characteristics for the total sample and stratified by study arm. Patients were 56.2% female, 54.2% white race, and with a mean (SD) age of 46.7 (20.0) years. Approximately half of the extractions were considered complex (47.7%).

**Table 1:** Patient characteristics (baseline and intervention periods, *N* = 12,924)

	**CDS** (*n* = 4997)	**CDS-E** (*n* = 3859)	**SP** (*n* = 4068)	**Total** (*N* = 12,924)
**Age**, mean (SD) min–max	46.6 (20.5) 16–99	48.3 (20.0) 16–100	45.4 (19.2) 16–97	46.7 (20.0) 16–100
**Gender**				
Female	2802 (56.1)	2126 (55.1)	2340 (57.5)	7268 (56.2)
Male	2195 (43.9)	1733 (44.9)	1728 (42.5)	5656 (43.8)
**Race**				
Asian	432 (8.7)	324 (8.4)	367 (9.0)	1123 (8.7)
Black	927 (18.6)	714 (18.5)	732 (18.0)	2373 (18.4)
American Indian	35 (0.7)	20 (0.5)	28 (0.7)	83 (0.6)
White	2666 (53.4)	2098 (54.4)	2244 (55.2)	7008 (54.2)
Other	114 (2.3)	88 (2.3)	95 (2.3)	297 (2.3)
More than one race	52 (1.0)	36 (0.7)	35 (0.9)	112 (0.9)
Unknown or not reported	772 (15.5)	589 (15.3)	567 (13.9)	1928 (14.9)
**Hispanic ethnicity**				
Hispanic	256 (5.1)	172 (4.5)	210 (5.2)	638 (4.9)
Not Hispanic	3276 (65.6)	2441 (63.3)	2998 (73.7)	8715 (67.4)
Unknown	1465 (29.3)	1246 (32.3)	860 (21.1)	3571 (27.6)
**Complex extraction at visit**				
Yes	2514 (50.3)	1819 (47.1)	1830 (45.0)	6163 (47.7)
No	2483 (49.7)	2040 (52.9)	2238 (55.0)	6761 (52.3)
**Medicaid insurance at visit**				
Yes	2263 (45.3)	1606 (41.6)	1938 (47.6)	5807 (44.9)
No	2734 (54.7)	2253 (58.4)	2130 (52.4)	7117 (55.1)

*CDS*, clinical decision support; *CDS-E*, clinical decision support with patient education; *SP*, standard practice. *N* (%) reported unless otherwise specified

### Provider characteristics

Table 2 shows the characteristics of providers. Providers were 57.1% female, 53.1% white race, and with a mean (SD) age of 43.7 (11.2) years. Most providers were dentists or periodontists (91.8%), while 8.2% were oral surgeons. Only a minority of providers were regularly prescribing opioids for tooth extractions, with just 26.5% of providers prescribing an opioid at 15% or more of their extraction procedures (suggesting that broader organizational efforts to reduce opioid prescribing had taken root). During the baseline period, 29.7% (2158/7275) of extractions had an opioid prescription.

**Table 2:** Provider characteristics

	**CDS** (*n* = 16)	**CDS-E** (*n* = 16)	**SP** (*n* = 17)	**Total** (*N* = 49)
**Age**, mean (SD) min–max	46.1 (12.4) 31–63	45.7 (12.0) 29–60	39.6 (8.7) 28–59	43.7 (11.2) 28–63
**Gender**				
Female	6 (37.5)	8 (50.0)	14 (82.4)	28 (57.1)
Male	10 (62.5)	8 (50.0)	3 (17.7)	21 (42.9)
**Race**				
Asian	1 (6.3)	1 (6.3)	3 (17.7)	5 (10.2)
Black	0 (0.0)	0 (0.0)	1 (5.9)	1 (2.0)
White	11 (68.8)	9 (56.3)	6 (35.3)	26 (53.1)
Unknown or not reported	4 (25.0)	6 (37.5)	7 (41.2)	17 (34.7)
**Provider type**				
Dentist, periodontist	14 (87.5)	15 (93.8)	16 (94.2)	45 (91.8)
Oral surgeon	2 (12.5)	1 (6.3)	1 (5.9)	4 (8.2)
**Baseline percentage of extractions with an opioid prescription**				
0– < 5	6 (37.5)	7 (43.8)	8 (47.1)	21 (42.9)
5–14	5 (31.3)	5 (31.3)	5 (29.4)	15 (30.6)
15–40	3 (18.8)	3 (18.8)	3 (17.7)	9 (18.4)
> 40	2 (12.5)	1 (6.3)	1 (5.9)	4 (8.2)

*CDS*, clinical decision support; *CDS-E*, clinical decision support with patient education; *SP*, standard practice. Age is known for 48 of 49 providers. *N* (%) reported unless otherwise specified

### Opioid prescribing (primary endpoint)

Table 3 shows odds ratios, model-based predicted probabilities, and confidence intervals for the main comparisons of interest. Additional file 1: Table S1 shows the full model parameters. Within each study arm, there was a significant decrease in opioid prescribing from the baseline to the intervention period. In the CDS arm, the odds of an extraction encounter resulting in an opioid prescription was 28% lower for the intervention period compared to the baseline period (*P* < 0.001), representing a reduction in the model-predicted probability of an opioid prescription from 18.6 to 14.1%. Likewise, there was a similar reduction from the baseline to the intervention period in the CDS-E arm (31% reduction in odds; *P* = 0.002), with the predicted probability of an opioid prescription falling from 14.8 to 10.7%. However, the SP arm also showed a reduction in opioid prescribing from the baseline to the intervention period, whereby the odds of an opioid prescription were reduced by 44% (*P* < 0.001)—a decrease in the predicted probability of opioid prescription from 16.4 to 9.8%.

**Table 3:** Model results for same-day opioid prescription

	**Model-derived estimates** **of same-day opioid prescription, % (95% CI)**			**OR**_**CDS (I vs B)**_**/** **OR**_**SP (I vs B)**_		**OR**_**CDS-E (I vs B)**_**/** **OR**_**SP (I vs B)**_	
	CDS	CDS-E	SP	OR (97.5% CI)	p	OR (97.5% CI)	p
Baseline	(*n* = 2728)	(*n* = 2199)	(*n* = 2348)				
	18.6 (12.8, 26.3)	14.8 (9.5, 22.3)	16.4 (10.8, 24.0)				
Intervention	(*n* = 2269)	(*n* = 1660)	(*n* = 1720)				
	14.1 (9.5, 20.6)	10.7 (6.7, 16.7)	9.8 (6.2, 15.2)				
OR, I vs B (95% CI)	0.72 (0.60, 0.86)	0.69 (0.55, 0.86)	0.56 (0.45, 0.70)	1.29 (0.93, 1.80)	.080	1.24 (0.86, 1.79)	.184
*p* (I vs B)	< .001	.002	< .001				

*CDS*, clinical decision support; *CDS-E*, clinical decision support with patient education; *SP*, standard practice; *OR*, odds ratio; *CI*, confidence interval; *I*, intervention period; *B*, baseline period. Generalized linear mixed model with fixed effects of study arm, time, study arm by time, baseline prescribing strata (includes provider type), complex extraction indicator, patient sex, patient age, and random intercept for provider

Comparison of differential change across arms showed no significant differences comparing the CDS and SP arms (OR = 1.29; *P* = 0.08) or the CDS-E and SP arms (OR = 1.24; *P* = 0.18). While differences were non-significant, decreases in opioid prescribing numerically favored the SP arm. Thus, there appeared to be a broader trend to de-implement the use of opioids for post-extraction pain, which was reflected in all three study arms.

### Analyses excluding oral surgeons

Because oral surgeons were more likely than general dentists to prescribe opioids in both the baseline and intervention periods, the analyses were re-run excluding the 4 oral surgeons in the sample (Additional file 2: Table S2). This did not appreciably alter conclusions with respect to comparisons between study arms (i.e., CDS vs. SP and CDS-E vs. SP comparisons remained non-significant). Within the CDS arm, the decrease in opioid prescribing from the baseline to the intervention period no longer met the statistical significance threshold at the 0.05 level (OR = 0.73; *P* = 0.07).

### CDS utilization

Ongoing examination of fidelity to the intervention revealed that many providers did not utilize the CDS, even when it was made available to them, despite awareness-raising activities and trainings by the study team. Oral surgeons were unlikely to utilize the CDS. There were three oral surgeons assigned to a CDS arm (2 to CDS, 1 to CDS-E), and they used the CDS at just 0.5% of extractions. There was wide variability across providers, with providers using the CDS between 0 and 87.4% of their extraction encounters. This includes 12.1% who never opened the CDS, 39.4% who used the CDS for fewer than 20% of their extraction encounters, 36.4% who used the CDS for between 21 and 50% of their extraction encounters, and 12.1% who used the CDS for 51–87% of their extraction encounters.

Table 4 shows the relationship between opioid prescribing and CDS utilization for non-oral surgeon providers in the CDS and CDS-E arms, stratified by extraction complexity. (Oral surgeons were excluded from this analysis because they accessed the CDS for only 10 extraction encounters.) In this sample, the CDS was opened for 29.9% of complex extractions and 22.0% of non-complex extractions. The association between CDS utilization and opioid prescribing was not significant for both complex (OR = 1.74; *P* = 0.09) and non-complex extractions (OR = 1.99; *P* = 0.14).

**Table 4:** Same-day opioid prescribing by CDS use, stratified by complex extraction status (*N* = 2055 patients linked to 29 non-oral surgeon dentists)

	**Model-derived estimates of same-day opioid prescription, % (95% CI)**		**OR**_**CDS opened vs. not opened**_	
Complex extractions (*n* = 598)	CDS opened (*n* = 179)	CDS not opened (*n* = 419)	OR (95% CI)	*p*
	16.6% (9.4, 27.6)	10.2% (6.2, 16.4)	1.74 (0.91, 3.31)	.09
Non-complex extractions (*n* = 1457)	CDS opened (*n* = 321)	CDS not opened (*n* = 1136)	OR (95% CI)	*p*
	1.8% (0.8, 4.1)	0.9% (0.5, 1.8)	1.99 (0.78, 5.05)	.14

*CDS*, clinical decision support; *OR*, odds ratio; *CI*, confidence interval. Analysis is restricted to *N* = 2055 patients linked to 29 non-oral surgeon dentists in CDS and CDS-E arms during the intervention period. Oral surgeons are excluded due to their very low level of CDS utilization (accessed CDS for 10 extractions). Generalized linear mixed model with fixed effects of baseline prescribing strata, patient sex, patient age, CDS opened, complex extraction indicator, interaction of CDS opened and complex extraction indicator, and random intercept for provider

## Discussion

In this multi-clinic, cluster randomized trial of clinical decision support strategies to de-implement opioid prescribing for dental extractions, we found that equipping dentists with clinical decision support did not lead to greater reductions in opioid prescribing compared to standard practice. Likewise, the inclusion of patient education materials in tandem with the provider’s clinical decision support did not lead to greater reductions in opioid prescribing. An important context for these findings is that opioid prescribing was already experiencing steady declines in the health system that hosted the study. As an organization, HealthPartners was already paying close attention to opioids and undertaking several actions to reduce opioid prescribing by providers. This was evidenced by a substantial reduction in opioid prescribing from the baseline study period (measured prospectively for 12 months starting in January 2019) and practices in the prior year (which informed some of our design decisions surrounding power and sample size). Our findings are consistent with recent research indicating that patterns of opioid prescribing in dentistry are influenced by type of extraction and that opioid prescribing overall has decreased in recent years in the wake of national practice guidelines and state legislation [8, 15, 21].

Another important context is the COVID-19 pandemic, which resulted in a significant disruption to dental care and may have impacted workflow even after the resumption of routine services. COVID-19 shutdowns began early during the intervention period. Although clinics re-opened soon after the start of the pandemic, services were limited to addressing urgent or emergency needs in certain clinics by a limited number of providers. After several months, the clinics transitioned back to normal operations while adjusting to new workflows and dealing with anxious patients. Dentists adjusting to the challenges of delivering care in the era of COVID possibly paid less attention to the CDS resources than they would have under normal circumstances.

The higher volume of opioid prescribing among oral surgeons in our sample was expected and consistent with prior literature. Research has found that the distribution of opioid prescribing by dentists differs markedly across provider characteristics, and a small minority of dentists account for high-volume and high-risk prescriptions, such as specialists in oral and maxillofacial surgery, who tend to perform more complex procedures [22]. This reflects what we found in the current study. What was less expected is that the oral surgeons were unlikely to use the CDS resources made available to them at all. Utilization of the CDS was low in this study, but especially so among oral surgeons, who were most likely to prescribe opioids and thus could stand to benefit most from the CDS. A recent systematic review of studies examining computerized clinical decision support systems found that uptake of such systems was generally low, and often not reported in trials [23]. Thus, our findings are consistent with prior work, which has found limited utilization of CDS among healthcare providers.

There is evidence that when utilized, CDS resources can change provider behavior [24, 25]. However, our study did not find utilization of the CDS to be significantly associated with opioid prescribing, and the likelihood of prescribing an opioid was numerically (albeit non-significantly) higher among dentists that accessed the CDS for a given encounter. It is possible that dentists were more likely to access the CDS when they were already considering prescribing an opioid and used it for confirming their decision. In such cases, the CDS could have informed providers’ discussions with patients about risk mitigation, even if the provider went forward with prescribing an opioid. It is also possible that our classification of extractions as complex and non-complex may have been insufficiently nuanced to fully account for meaningful differences in patients’ extractions. It is unknown whether broader uptake of the CDS could have impacted provider behavior.

Although dentistry and the healthcare system as a whole are moving away from opioid prescribing, it is important to recognize that opioids can still play an important role for some patients and may be appropriate in certain circumstances [26]. Nevertheless, there is a consensus that, as in the healthcare system more broadly, dentistry has been overly reliant on opioids for managing post-extraction dental pain. While there is good evidence that ibuprofen/acetaminophen combination is effective for managing post-extraction pain, research in this area is ongoing, including a large multi-clinic, double-blind non-inferiority trial that will directly compare a non-opioid NSAID combination (ibuprofen/acetaminophen) against the most commonly used opioid analgesic combination product (hydrocodone/acetaminophen) for third molar extraction pain [27]. That study will examine a broader set of patient outcomes that could potentially influence oral surgeons’ reliance on opioids.

### Limitations

This study has several important limitations. As a real-world, practical de-implementation trial, the study was bound to a particular time and place, and thus subject to the influence of larger environmental and system-level factors such as broader trends in decreasing opioid prescribing and the disruptions associated with the COVID-19 pandemic. These factors complicate interpretation, insofar as it is unclear if our findings regarding CDS would generalize to a context in which opioid prescribing trends were steadier in standard practice, or during periods of time when health services were not in a state of upheaval and reorganization. To some extent, these challenges are inherent to all implementation research. Conducting this study in a single large multi-clinic health system offered important advantages with respect to feasibility, but also may limit generalizability. While randomizing at the provider level has various advantages for implementation science, it can be difficult to achieve balance on all relevant provider characteristics. Another limitation is that utilization of the CDS was low among providers randomized to CDS or CDS-E arms. However, the study team made reasonable efforts to ensure that the CDS was easily accessible and that providers were aware of this resource. It is nonetheless possible that the training did not adequately address hesitancy among providers or sufficiently convey the utility of the CDS for informing discussions with patients. If providers did not consider the CDS to be of value in their prescribing decisions, it is possible that other strategies (e.g., training on the most recent prescribing guidelines, tailored feedback, etc.) would be more successful in changing practices. Future research should focus on ways to increase the perceived relevance of CDS resources for providers, and investigate other approaches to de-implementing potentially harmful practices.

## Conclusions

This prospective, multi-clinic cluster randomized trial found broad trends towards de-implementing the use of opioids for pain management following dental extractions. Making CDS resources available to dental providers, either with or without patient education, did not lead to greater reductions in opioid prescribing relative to standard care, but utilization of CDS was generally low. Future research should track opioid prescribing practices and identify strategies that can promote the best evidence care.

## Supplementary Information

**Additional file 1:** **Ancillary Table 1.** Full model results forsame-day opioid prescription.

**Additional file 2:** **Ancillary Table 2.** Model results for same-day opioid prescription,excluding oral surgeons.

## Data Availability

The datasets used for the current study are available, pending approval of a research proposal and execution of a data-sharing agreement with HealthPartners.
