# Supplementary material for: De-Implementing Opioids for Dental Extractions (DIODE): a multi-clinic, cluster-randomized trial of clinical decision support strategies in dentistry
Source: Implement Sci. 2023 Feb 10;18:5. doi: 10.1186/s13012-023-01262-7 (PMC9913004; doi:10.1186/s13012-023-01262-7)
Supplement: Supplementary file 1 — Additional file 1: Ancillary Table 1. Full model results forsame-day opioid prescription. [file 13012_2023_1262_MOESM1_ESM.docx]

Ancillary Table 1. Full model results for same-day opioid prescription.

|  | OR (95% CI) | p |
| --- | --- | --- |
| Study Arm (ref = Standard Practice arm) | - | - |
| CDS arm | 1.33 (0.75, 2.36) | 0.588 |
| CDS-E arm | 0.99 (0.54, 1.80) | 0.691 |
| Time Period (ref = Baseline) |  |  |
| Intervention period | 0.65 (0.58, 0.74) | <0.001 |
| Study arm * Time interaction: CDS (I vs. B) vs. SP (I vs. B) | 1.29 (0.97, 1.72) | 0.080 |
| Study arm * Time interaction: CDSe (I vs. B) vs. SP (I vs. B) | 1.24 (0.90, 1.70) | 0.184 |
| Baseline opioid prescribing strata (ref = General dentists, prescribing 15%-40%) |  |  |
| Oral surgeons, prescribing > 40% | 3.90 (1.68, 9.05) | 0.002 |
| General dentists, prescribing 5-14% | 0.34 (0.18, 0.65) | 0.002 |
| General dentists, prescribing <5% | 0.06 (0.03, 0.12) | <0.001 |
| Complex extraction (ref = not complex) | 7.24 (6.34, 8.26) | <0.001 |
| Female patient (ref= Male) | 0.80 (0.72, 0.90) | <0.001 |
| Patient age at index visit (per 10 years) | 0.65 (0.63, 0.67) | <0.001 |

Notes. CDS = Clinical decision support, CDS-E = Clinical decision support with patient education, SP= Standard practice. OR= Odds ratio. CI= Confidence Interval. I= Intervention period. B= Baseline period. Generalized linear mixed model with fixed effects of study arm, time, study arm by time, baseline prescribing strata (includes provider type), complex extraction indicator, patient sex, patient age and random intercept for provider.
