# Supplementary material for: De-Implementing Opioids for Dental Extractions (DIODE): a multi-clinic, cluster-randomized trial of clinical decision support strategies in dentistry
Source: Implement Sci. 2023 Feb 10;18:5. doi: 10.1186/s13012-023-01262-7 (PMC9913004; doi:10.1186/s13012-023-01262-7)
Supplement: Supplementary file 2 — Additional file 2: Ancillary Table 2. Model results for same-day opioid prescription,excluding oral surgeons. [file 13012_2023_1262_MOESM2_ESM.docx]

Ancillary Table 2. Model results for same-day opioid prescription, excluding oral surgeons.

|  | **Model-derived estimates of same-day opioid prescription, % (95% CI)** | | | **OR_CDS (I vs B)_ /**  **OR_SP (I vs B)_** | | **OR_CDS-E (I vs B)_ /**  **OR_SP (I vs B)_** | | |
| --- | --- | --- | --- | --- | --- | --- | --- | --- |
|  | CDS | CDS-E | SP | OR  (97.5% CI) | p | OR  (97.5% CI) | p |  |
|  | (n=1,509) | (n=1,389) | (n=1,562) |  |  |  |  |  |
| Baseline  (95% CI) | 3.6  (2.5, 5.3) | 3.1  (2.1, 5.3) | 3.6  (2.5, 5.2) |  |  |  |  |  |
|  |  |  |  |  |  |  |  |  |
|  | (n=1,077) | (n=978) | (n=1,020) |  |  |  |  |  |
| Intervention  (95% CI) | 2.7  (1.7, 4.1) | 1.5  (0.9, 2.5) | 1.7  (1.1, 2.7) |  |  |  |  |  |
|  |  |  |  |  |  |  |  |  |
| OR, I vs B  (95% CI) | 0.73  (0.52, 1.03) | 0.47  (0.29, 0.75) | 0.47  (0.31, 0.70) | 1.56  (0.85, 2.88) | .096 | 1.00  (0.49, 2.05) | .999 |  |
| p (I vs B) | .070 | .002 | <.001 |  |  |  |  |  |

Note. CDS = Clinical decision support, CDS-E = Clinical decision support with patient education, SP = Standard Practice. OR= Odds ratio. CI= Confidence Interval. I= Intervention period. B= Baseline period. Analysis includes 45 general dentists and excludes four oral surgeons. Generalized linear mixed model with fixed effects of study arm, time, study arm by time, baseline prescribing strata, complex extraction indicator, patient sex, patient age and random intercept for provider.
